# Oxylipin Biomarkers of Auto-Oxidation Are Associated with Antioxidant Micronutrients and Multiple Sclerosis Disability

**DOI:** 10.3390/antiox15010102

**Published:** 2026-01-13

**Authors:** Taylor R. Wicks, Anna Wolska, Diala Ghazal, Irina Shalaurova, Bianca Weinstock-Guttman, Richard W. Browne, Alan T. Remaley, Robert Zivadinov, Murali Ramanathan

**Affiliations:** 1 Department of Pharmaceutical Sciences, University at Buffalo, The State University of New York, Buffalo, NY 14203, USA; trwicks@buffalo.edu; 2 Lipoprotein Metabolism Laboratory, National Heart, Lung, and Blood Institute, National Institutes of Health, Bethesda, MD 20892, USA; anna.wolska@nih.gov (A.W.);; 3 Biotechnical and Clinical Laboratory Sciences, University at Buffalo, The State University of New York, Buffalo, NY 14203, USA; 4 LabCorp Diagnostics, Morrisville, NC 27560, USA; 5 Jacobs Multiple Sclerosis Center, Department of Neurology, Jacobs School of Medicine and Biomedical Sciences, University at Buffalo, The State University of New York, Buffalo, NY 14203, USA; bw8@buffalo.edu; 6 Buffalo Neuroimaging Analysis Center, Department of Neurology, Jacobs School of Medicine and Biomedical Sciences, University at Buffalo, The State University of New York, Buffalo, NY 14203, USA; 7 Center for Biomedical Imaging, University at Buffalo, The State University of New York, Buffalo, NY 14203, USA

**Keywords:** lipid, polyunsaturated fatty acids, omega fatty acids, oxidative stress

## Abstract

**Purpose:** To investigate associations between lipid oxidation biomarkers (oxylipins), antioxidant micronutrients, lipoprotein particles, and apolipoproteins in multiple sclerosis (MS). **Methods:** Blood and neurological assessments were collected from 30 healthy controls, 68 relapsing remitting MS subjects, and 37 progressive MS subjects. Hydroxy (H) and hydroperoxy lipid peroxidation products of the polyunsaturated fatty acids (PUFAs) arachidonic (20:4, ω-6), linoleic (octadecadienoic acid or ODE, 18:2, ω-6), eicosapentaenoic (20:5, ω-3), and α-linolenic (18:3, ω-3) acids were measured using liquid chromatography–mass spectrometry. Antioxidant micronutrients, including β-cryptoxanthin and lutein/zeaxanthin, were quantified by high-performance liquid chromatography. Lipoprotein and metabolite profiles were obtained using nuclear magnetic resonance spectroscopy. Regression models were adjusted for age, sex, body mass index, and disease status. **Results:** The 9-hydroxy octadecadienoic acid to 13-hydroxy octadecadienoic acid ratio (9-HODE/13-HODE ratio), which reflects autoxidative versus enzymatic oxidation, was associated with MS status (*p* = 0.002) and disability on the Expanded Disability Status Scale (*p* = 0.004). Lutein/zeaxanthin (*p* = 0.023) and β-cryptoxanthin (*p* = 0.028) were negatively associated with the 9-HODE/13-HODE ratio. Apolipoprotein-CII, a marker of liver-X-receptor (LXR) signaling, was associated with 9-HODE/13-HODE ratio and other oxylipins. Octadecadienoic fatty acid-derived oxylipins were negatively associated with LC3A, a mitophagy marker, and positively correlated with 7-ketocholesterol, a cholesterol autoxidation product. **Conclusions:** Autoxidation of PUFAs is associated with greater disability in MS. Higher β-cryptoxanthin and lutein/zeaxanthin were associated with reduced auto-oxidation. Lipid peroxidation shows associations with LXR signaling, mitophagy, inflammation, and cholesterol autoxidation.

## 1. Introduction

Multiple sclerosis (MS) is a chronic neurodegenerative disease affecting the brain and spinal cord, characterized by progressive physical and cognitive disability [1,2,3]. Blood–brain barrier breakdown, inflammation, demyelination, and neurodegeneration are the central pathophysiological mechanisms in MS. Oxidative stress is a central metabolic mechanism that can cause neurodegeneration.

Oxidative stress is an imbalance between the damaging effects of reactive oxygen species (ROS) and the detoxifying effects of antioxidant defense molecules that eliminate or scavenge ROS and repair damaged biomolecules.

**Figure 1:**
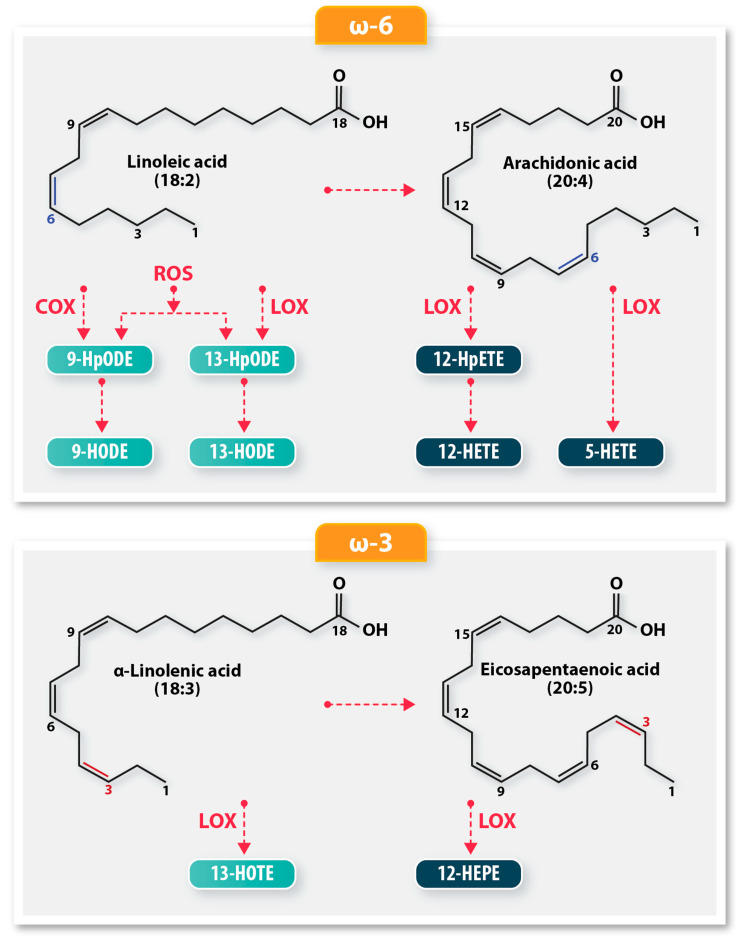
A schematic of the biochemical pathways resulting in oxylipin derivatives from polyunsaturated fatty acids (PUFA). The ω-6 PUFA, linoleic acid, is oxidized by lipoxygenases (LOX), cyclooxygenases (COX), or autoxidation to produce 9-HODE and 13-HODE. LOX oxidation produces more 13-HODE, while COX oxidation produces a preponderance of 9-HODE oxylipins. Auto-oxidation produces equal amounts of 9-HODE and 13-HODE. Arachidonic acid utilizes different LOX enzymes to produce oxylipin derivatives 12-HpETE, 12-HETE, and 5-HETE. The ω-3 fatty acids, α-linolenic acid and eicosapentaenoic acid, are enzymatically oxidized by LOX to produce the hydroxy oxylipins, 13-HOTE and 12-HEPE, respectively.

Polyunsaturated fatty acids (PUFAs) in lipids are a common target of ROS, leading to lipid peroxidation, a free radical chain reaction, producing hydroperoxy-derivatives. Hydroperoxides are rapidly converted to less reactive hydroxy derivatives by cellular peroxidases. As a group, these hydroperoxy and hydroxy PUFA oxidation products are known as oxylipins (Figure 1). PUFAs can also be oxidized to oxylipins by enzymes such as cyclooxygenases (COX) and lipoxygenases (LOX), including COX-1, COX-2, 15-LOX-1, and 15-LOX-2. In the presence of transition metals like iron, lipid hydroperoxides can form reactive aldehydes that can react with proteins [4]. The continued oxidation of PUFAs can result in altered lipoprotein structure and function, cellular membrane integrity loss, and inactivation of membrane-bound proteins, triggering apoptosis, necrosis, ferroptosis, and inflammatory effects [5,6].

The 9-HODE/13-HODE ratio is an important indicator of lipid peroxidation, as it reflects the relative contributions of auto-oxidation, COX, and LOX mechanisms to the oxidative stress environment [7]. 9-HODE/13-HODE ratio values near 1 indicate autoxidation, values greater than 1 reflect predominant COX activity, and values less than 1 are indicative of LOX activity.

Increased dietary intake of ω-3 fatty acids, α-linolenic acid (ALA), eicosapentaenoic acid (EPA), and docosahexaenoic acid (DHA) are associated with lower levels of pro-inflammatory mediators, C-reactive protein (CRP), and GlycA [8], and with lower incidence of cardiovascular diseases [7], which have been linked to MS progression [9]. ω-6 fatty acids are thought to exert a negative effect on human health.

The balance between anti-inflammatory properties of ω-3 and pro-inflammatory properties of ω-6 PUFAs could also be clinically relevant, given the central role of neuroinflammation in MS pathogenesis. The correlation between fish consumption and lower MS prevalence, [9,10,11] as well as slower disease progression [12,13] in epidemiological studies, can be plausibly attributed to the vitamin D and ω-3 polyunsaturated fatty acid content in fish. A diet high in ω-3 over ω-6 PUFAs has been suggested to be crucial for bodily processes and overall health in MS. A review of 5554 studies on the effects of EPA, DHA, and docosapentaenoic acid (DPA) demonstrated increased ω-3 and fish oil supplementation was beneficial in improving quality of life and disability [10], and reduced relapse rates [14] in patients with MS (pwMS). Excessive consumption of ω-6 PUFAs correlated with increased MS incidence [15]. While ω-6 fatty acids such as arachidonic acid (ARA) may be a precursor for inflammation [16], there are potential benefits of ω-6 PUFA intake. Long-term mixed intake of ω-6 and ω-3 fatty acids improved gait and functional capacity in relapsing-remitting MS patients (RRMS) [17], and total ω-6 PUFAd levels contributed to a decreased MS risk [18]. However, interventional studies with fish oil, ω-3 [19], and ω-6 [20] polyunsaturated fatty acid supplementation showed no positive effects on MS clinical outcomes [19,21].

Dietary carotenoids, retinols, and provitamins are potent antioxidants that can reduce oxidative damage by scavenging and terminating free radical-driven chain reactions. Trials of antioxidants as disease-modifying treatments (DMT) in MS have yielded limited and conflicting effects [22,23].

Lipid peroxidation is thought to be a significant contributor to MS inflammation [24] and disease development [25] through oxylipin signaling. The central premise therefore was to investigate whether the products and mechanistic processes (enzymatic versus auto-oxidative) of oxidation, rather than the PUFAs themselves, are relevant to the progression of MS. Determining the involvement of oxylipins in MS could provide mechanistic insight into the failure of fish oil and PUFA supplementation trials. The objective of the research was to investigate the mechanistic contributions, if any, of oxylipins, with antioxidants, lipoproteins, apolipoproteins, and chronic inflammation to MS disability.

## 2. Materials and Methods

### 2.1. Study Design

Study setting and design: This single-center, cross-sectional exploratory study was performed at the Jacobs Multiple Sclerosis Center for Treatment and Research of the University at Buffalo, an academic MS center in Buffalo, NY, USA.

Informed consent: The study protocol was approved by the University at Buffalo Human Subjects Institutional Review Board (Approval code: MODCR00009015), and written consent was obtained from all patients in line with the Declaration of Helsinki.

Clinical assessment: Demographic information was documented, and neurological assessments were performed on all subjects.

Healthy controls (HC) were included if they had a normal examination and were free of neurological disease. Exclusion criteria for HC were preexisting medical conditions associated with brain pathology, including cerebrovascular disease and alcohol abuse disorders.

MS disease course was diagnosed by a licensed neurologist utilizing the 2010 revision of the McDonald criteria. The inclusion criteria for participants with MS for the sub-study included age ≥ 18 and the availability of one or more oxylipin markers as well as NMR biomarkers. The exclusion criteria were: presence of a relapse, use of corticosteroids within 30 days prior to the study, history of cerebral congenital vascular malformations, contraindications for MRI contrast agents, and pregnant or nursing mothers.

The progressive MS (PMS) group consisted of secondary-progressive MS (SPMS) and primary-progressive MS (PPMS) subtypes. The Expanded Disability Status Scale (EDSS) was used as a measure of disability.

### 2.2. Serum Biomarker Analysis

#### 2.2.1. Serum Analysis

Non-fasting blood samples from all participants were separated into serum and plasma within 24 h, and frozen in aliquots at −80 °C. The clinical samples were de-identified for analysts.

#### 2.2.2. Lipid Peroxidation Products Measurements

Total monohydroxy (H) and monohydroperoxy (Hp) lipid peroxidation products of eicosatetraenoic (ETE, 20:4 or arachidonic acid), octadecadienoic (ODE, 18:2 or linoleic acid), and octadecatrienoic (OTE, 18:3 or linolenic acid) fatty acid species were measured from serum samples precisely as described by Zhu et al. [26]. The fatty acids standards; ((±)-9-hydroxy-10E,12Z-octadecadienoic acid (9-HODE), (±)-13-hydroxy-9Z,11E-octadecadienoic acid (13-HODE), 9-hydroperoxy-10E,12Z-octadecadienoic acid (9-HpODE), (±)13-hydroperoxy-9Z,11E-octadecadienoic acid (13-HpODE), 13S-hydroxy-9Z,11E,15Z-octadecatrienoic acid (13(s)-HOTrE), ±12-hydroxy-5Z,8Z,10E,14Z,17Z-eicosapentaenoic acid (12-HEPE), (±)5-hydroxy-6E,8Z,11Z,14Z-eicosatetraenoic acid (5-HETE), (±)12-hydroxy-5Z,8Z,10E,14Z-eicosatetraenoic acid (12-HETE)), and deuterated internal standards 12S-hydroxy-5Z,8Z,10E,14Z-eicosatetraenoic-5,6,8,9,11,12,14,15-d8 acid (12(s)-HETE-d8) were purchased from Cayman Chemical (Ann Arbor, MI). Oxylipin concentrations were expressed in ng/mL.

The 9-HODE/13-HODE ratio was computed as a measure of auto-oxidative vs. enzymatic activation. A ratio closer to 1 indicates a more equal balance between auto-oxidation and enzymatic oxidation.

#### 2.2.3. Antioxidants

Retinols, tocopherols, and carotenoids collected from serum samples consisted of α-carotene, β-carotene, α-tocopherol, δ-tocopherol, γ-tocopherol, β-cryptoxanthin, lutein/zeaxanthin, and lycopene. Calibrators were prepared in ethanol or hexane, and their absorbance was measured spectrophotometrically using the absorptivity coefficients provided by the National Institute of Standards and Technology (NIST). All coefficients of variation were <15%. Antioxidant concentrations were expressed in µg/mL.

The analytes were assayed by high-performance liquid chromatography (HPLC) using previously published methods, validated in accordance with Food and Drug Administration guidelines, and quality assured through the NIST Micronutrient Measurement Quality Assurance Program [27].

#### 2.2.4. Nuclear Magnetic Resonance (NMR) Spectroscopy

NMR spectroscopy was used to measure lipoproteins particle number and sizes (LabCorp, Morrisville, NC, USA) in serum samples analyzed by the LP4 algorithm [28]. The algorithm quantified lipoprotein subclasses of different sizes: low-density lipoprotein (LDL) particles (LDLP), triglyceride-rich lipoprotein (TRL) particles (TRLP), and high-density lipoprotein (HDL) particles (HDLP).

The HDL particle subspecies provided were H1P (diameter: 7.4 nm), H2P (7.8 nm), H3P (8.7 nm), H4P (9.5 nm), H5P (10.3 nm), H6P (10.8 nm), and H7P (12.0 nm), and were further categorized into small (H1P + H2P), medium (H3P + H4P), and large-HDLP (H5P + H6P + H7P).

In addition, the signal from glycan residues of acute-phase glycoproteins, known as GlycA, was measured as an NMR-derived biomarker of inflammation.

Lipoprotein particle diameters were presented in nm, TRLP and LDLP concentrations were expressed in nM; HDLP and GlycA concentrations were expressed in µM.

#### 2.2.5. Apolipoproteins

Apolipoprotein (Apo), Apo-AI, Apo-AII, ApoB, ApoC-II, and ApoE, levels were measured with immunoturbidometric diagnostic reagent kits, calibrators, and quality control materials (Kamiya Biomedical, Thousand Oaks, CA, USA) as previously published [29]. Apolipoprotein concentrations were expressed in mg/dL.

#### 2.2.6. Neurofilament Light Chain (NfL)

Serum NfL (sNfL) was measured using a single-molecule array (SIMOA) immunoassay through a collaboration with the University of Basel. sNfL concentrations are in pg/mL.

#### 2.2.7. Cholesterol Auto-Oxidation

As a measure of cholesterol autooxidation, 7-ketocholesterol (7-KC) was measured in EDTA plasma using low-temperature saponification, solid-phase extraction, and LC-MS analysis as previously described [30]. 7-KC concentration was expressed in ng/mL.

#### 2.2.8. Mitophagy Marker

Microtubule-associated protein 1A/1B light chain 3 alpha (LC3A), a structural protein of autophagosomes, was measured in serum samples using an immunoassay (R&D Systems, Minneapolis, MN, USA). LC3A concentration was expressed in ng/mL.

#### 2.2.9. C-Reactive Protein

CRP was measured with the high-sensitivity assay (hs-CRP) on the ABX Pentra 400 automated chemistry analyzer (Horiba Medical, Montpellier, France).

### 2.3. Data Analysis

All statistical analyses were conducted with the R (4.1.2) statistical computing program; plots were generated with the ggplot2 package.

Oxylipins, apolipoproteins, and NMR biomarkers were logarithm (base 10) transformed for regression analysis. Oxylipins were treated as predictor variables, and age, sex, BMI, and the individual biomarker measure of interest as dependent variables in the regression analysis.

The rstatix R package was used to compute the generalized eta-squared (a measure of effect size, *η*^2^) and predictor *p*-values from regression analyses [31]. Small, medium, or large effect sizes of *η*^2^ thresholds are ≥0.01, 0.06, and 0.14, respectively.

## 3. Results

### 3.1. Clinical and Demographic Characteristics

Table 1 summarizes the demographic and clinical characteristics of the study groups. The PMS group had higher EDSS scores, was older, and had a longer disease duration than the RRMS group, which reflects the individualized disease courses.

### 3.2. Oxylipin Biomarkers of Lipid Peroxidation in MS

**Figure 2:**
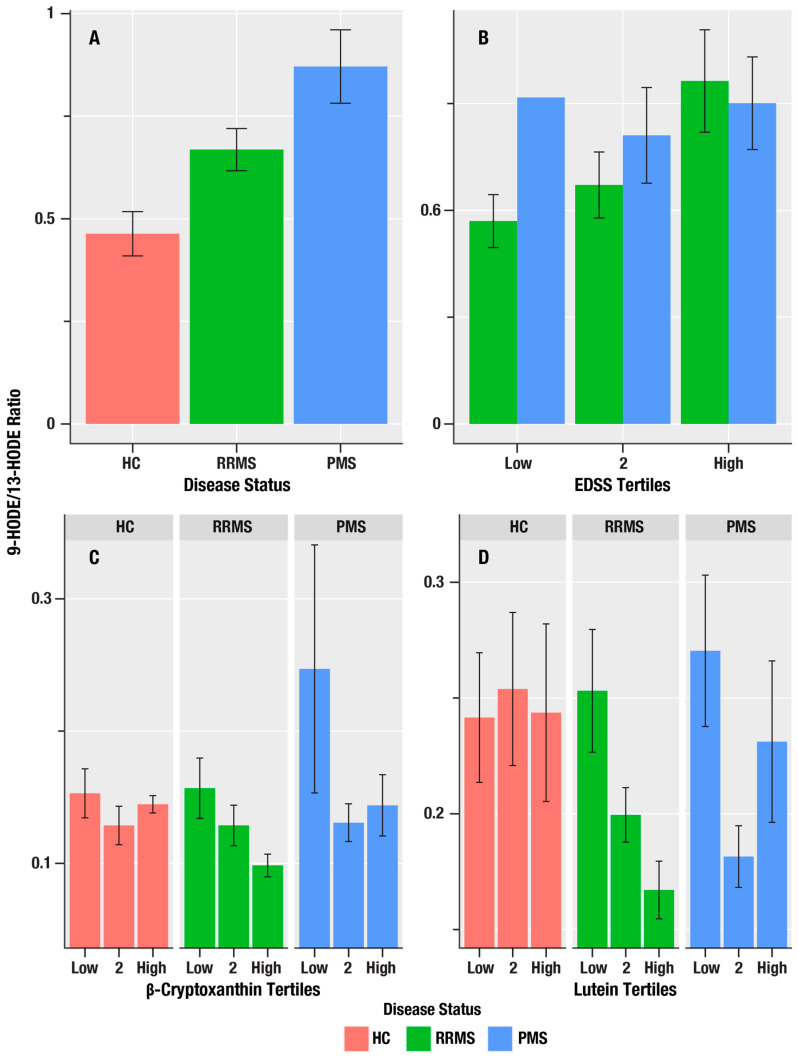
The associations of MS disease course status, disability, and antioxidant micronutrients on 9-HODE/13-HODE oxylipin ratio in HC, RR, and PMS Groups. (**A**) is a bar graph of the mean 9-HODE/13-HODE ratio in healthy controls (HC, salmon bars), relapsing remitting MS (RRMS, green bars), and progressive MS (PMS, blue bars). (**B**) shows the mean 9-HODE/13-HODE ratio (*y*-axis) for the lowest, middle, and highest tertiles of disability on the Expanded Disability Severity Scale (EDSS) score and the 9-HODE/13-HODE ratio in RRMS (green bars), and PMS (blue bars). (**C**,**D**) show the mean 9-HODE/13-HODE ratio (*y*-axis) associations for the lowest, middle, and highest tertiles of the antioxidant micronutrients, β-cryptoxanthin and lutein/zeaxanthin, in HC (salmon bars), RRMS (green bars), and PMS (blue bars). The error bars are standard errors; the *p*-values from the linear regression analyses are in Table 2 for (**A**,**B**), and Table 3 for (**C**,**D**).

Table 2 summarizes associations of the oxylipin markers, 9-HODE, 13-HODE, 9-HpODE, 9-HODE/13-HODE ratio, 13-HOTE, 12-HEPE, 5-HETE, 12-HETE, and 12-HpETE, with markers of MS disease course (HC-RR-PMS status), disability (EDSS), and neuroaxonal injury (sNfL). The 9-HODE/13-HODE ratio, which reflects the ratio of auto-oxidation to enzymatic oxidation, was greatest in the PMS group and lowest in the HC group (i.e., in the order PMS > RR > HC), and was positively correlated with EDSS scores (*β* = 1.19, *η*^2^ = 0.094, *p* = 0.004; Figure 2). We did not obtain evidence for associations of oxylipins with sNFL.

To further corroborate the role of auto-oxidation in the associations of the 9-HODE/13-HODE ratio with the MS disease status, we assessed 7-ketocholesterol (7KC), which is known to result from cholesterol auto-oxidation. 7KC was positively associated with 9-HODE (*β* = 0.076, *η*^2^ = 0.051, *p* = 0.025) and 13-HODE (*β* = 0.097, *η*^2^ =0.053, *p* = 0.022), and its levels showed an increasing trend in the order PMS > RR > HC.

### 3.3. Antioxidant Micronutrients Are Associated with Lower Oxylipin Levels

Retinol, tocopherols, and carotenoids (RTC) are important fat-soluble vitamins and micronutrients with antioxidant properties that could inhibit lipid peroxidation to reduce oxylipin levels. Table 3 summarizes the regression results for the antioxidant micronutrients, α- and β-carotene, α-, γ-, and δ-tocopherol, β-cryptoxanthin, lutein/zeaxanthin, and lycopene, with oxylipins.

9-HODE, 13-HODE, 9-HpODE, 12-HEPE, 5-HETE, and 12-HpETE were associated with γ-tocopherol. 13-HpODE and 13-HOTE correlated with α-tocopherol.

**Figure 3:**
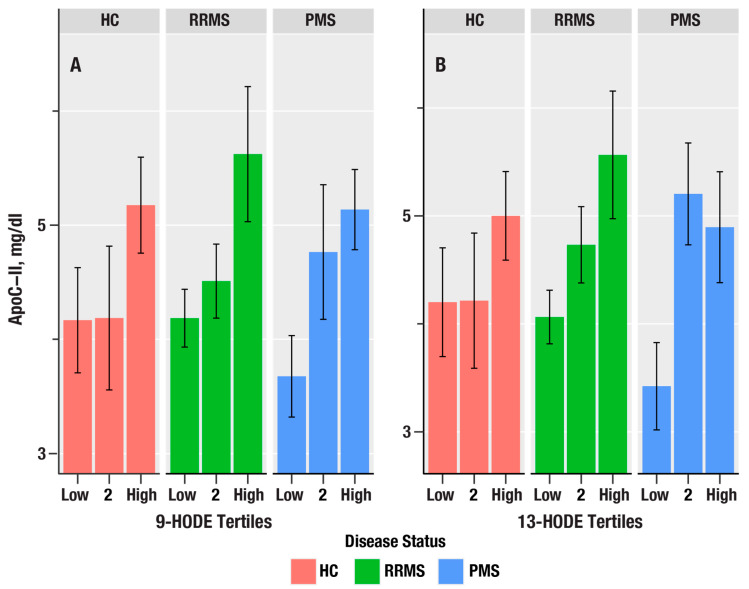
The bar plots in (**A**,**B**) show the dependence of apolipoprotein C-II (ApoC-II in mg/dL) on tertiles of 9-HODE and 13-HODE in healthy controls (HC, salmon bars), relapsing remitting MS (RRMS, green bars), and progressive MS (PMS, blue bars). The bars represent mean values, and the error bars are standard errors. The *p*-values from linear regression are in Table 4.

The 9-HODE/13-HODE ratio was associated with β-cryptoxanthin (*η*^2^ = 0.040, *p* = 0.028) and lutein/zeaxanthin (*η*^2^ = 0.043, *p* = 0.023; Figure 3).

### 3.4. Associations of Oxylipins with Lipoprotein Particle Size Subclasses

Lipoproteins are critical for the distribution of lipid peroxidation substrates, oxylipins, and diverse lipid nutrients. We examined the associations between oxylipins and lipoproteins to identify candidate lipoprotein particle size subclasses. The associations of individual oxylipins with the MS disease status (HC-RR-PMS), as estimated from regression analyses adjusted for age, sex, and body mass index (BMI), are shown in Table 4.

Triglyceride-rich lipoproteins: Chylomicrons, very low-density (VLDL), and intermediate-density lipoproteins (IDL) comprise the triglyceride-rich lipoprotein class. Table 4 summarizes the associations between TRL particle size subsets and oxylipins. Very small TRL (VS-TRL), which correspond to IDL, were associated with 9-HODE (*p* < 0.001), 13-HODE (*p* = 0.049), 9-HODE/13-HODE ratio (*p* = 0.005), 12-HEPE (*p* = 0.046), and 5-HETE (*p* = 0.038).

Since triglyceride levels are increased by liver X receptor (LXR) activation, we examined the associations of oxylipins with ApoC-II, an apolipoprotein biomarker induced by LXR [32]. 9-HODE, 13-HODE, 9-HpODE, 5-HETE, and 12-HpETE were positively associated with ApoC-II and increased from PMS > RR > HC. In contrast, ApoE, which is a key apolipoprotein expressed on TRL, was not associated with any of the oxylipins.

Low-density lipoproteins: Seven of the nine oxylipins were positively correlated with overall LDL particle (LDLP) size (Table S1). 5-HETE was associated with medium LDLP.

13-HODE, 9-HpODE, 13-HpODE, 12-HEPE, 5-HETE, and 12-HpETE were positively associated with ApoB, which is the characteristic apolipoprotein of LDL (Table S1).

High-density lipoproteins: Associations with high-density lipoprotein particles (HDLP) can be found in the Supplementary Materials (Table S2). 9-HODE and 13-HODE were negatively associated with large HDLP. There were no correlations between oxylipins and ApoA-I or ApoA-II.

### 3.5. Association of Chronic Inflammation and Mitophagy Biomarkers

C-reactive protein (CRP) and GlycA, which are established biomarkers of chronic inflammation, were measured using high-sensitivity immunoassay and NMR spectroscopy. GlycA levels correspond to glycan modifications on proteins induced by inflammation; CRP is induced by cytokines such as interleukin-6 and interleukin-1β. CRP and GlycA were positively associated with both 9-HODE and 13-HODE (See Table 5).

Mitochondria are a key intracellular source of ROS. Mitophagy is an active clearance process that maintains mitochondrial homeostasis. LC3A, a structural protein of autophagosomes that is a biomarker of mitophagy, was negatively associated (See Table 5) with 9-HODE (β = −0.028, *p* = 0.041), 13-HODE (β = −0.049, *p* = 0.004), 9-HpODE (β = −0.122, *p* < 0.001), and 13-HpODE (β = −0.122, *p* < 0.001). We did not obtain evidence for an association between LC3A and the 9-HODE/13-HODE ratio in the regression analysis that adjusted for disease status. These results indicate that increased mitophagy is associated with lower auto-oxidation and oxylipin levels.

## 4. Discussion

We investigated the associations of oxylipins with disability, antioxidants, and lipoprotein particle subclasses in MS. We found that a high 9-HODE/13-HODE ratio was associated with HC-RRMS-PMS status and EDSS. VS-TRL particles and antioxidants, β-cryptoxanthin and lutein/zeaxanthin, were correlated with the 9-HODE/13-HODE ratio. LA-derived oxylipins were negatively associated with LC3A and positively associated with 7-KC.

A strength of our study was the availability of diverse oxylipin profiles. We utilized a sophisticated multimodal analytical strategy combining LC-MS and HPLC assays for oxylipins and antioxidant micronutrients, respectively, NMR spectroscopy for detailed lipoprotein particle analysis, and immunoassays for signaling proteins. The LC-MS and HPLC assays for oxylipins [33,34] and antioxidant micronutrients [27,35] were validated in accordance with FDA guidelines, and we have employed them in population [36], epidemiological, and clinical studies, as well as in animal experiments [37]. Likewise, our NMR spectroscopy method has been used in large clinical studies of cardiovascular disease [38]. Limitations to the study include the single-center design and modest sample size. Given the exploratory nature of the study, we did not adjust for multiple testing.

Oxidative stress is a driver of pathological processes such as mitochondrial dysfunction, apoptosis, neurodegeneration, and tissue ageing [39]. Myelin is more susceptible to oxidative stress because it is rich in PUFAs, and the brain requires substantial oxygen uptake to maintain its high metabolic rate. The presence of high levels of oxidized lipids in pre-phagocytic lesions implicates oxidative stress at the earliest steps in MS lesion formation. Oxidatively modified LDL, malondialdehyde, and 4-hydroxynonenal, which are important, toxic products of lipid peroxidation, are prominent in early and actively demyelinating MS lesions [40]. Oxidative injury to oligodendrocytes and neurons occurs during demyelination and axonal injury in MS [41]. Plasma biomarkers, including plasma fluorescent lipid peroxidation products [42,43], total conjugated dienes [44], antibodies to oxidized LDL, [45] and F2-isoprostanes, [46,47,48] are also increased in MS. There is, however, a significant knowledge gap in our understanding of the mechanisms causing lipid peroxidation and the role of antioxidants in MS, due to the lack of integrated data on both primary oxidatively damaged products and antioxidant factors.

Hakansson et al. found higher CSF levels of 9-HODE and 13-HODE in MS patients compared to healthy controls, but did not find evidence for prognostic associations with disease activity at 2 or 4 years [49]. Auto-oxidation of linoleic acid by reactive oxygen species free radicals tends to be nonspecific and produces nearly equal amounts of 9-HODE and 13-HODE, resulting in a 9-HODE/13-HODE ratio of approximately 1. Enzymatic oxidation of linoleic acid by cyclooxygenase (COX) 1 and COX-2 enzymes predominantly results in 9-HODE; COX-1 and COX-2 also catalyze the conversion of arachidonic acid to prostaglandins. 13-HODE is the major product when linoleic acid is oxidized by 15-LOX-1; however, 15-LOX-2, COX-1, COX-2, and cytochrome P450 enzymes produce 13-HODE from linoleic acid. Thus, COX activities, which favor the production of 9-HODE, shift the 9-HODE/13-HODE ratio to greater than 1, and the LOX activities shift the 9-HODE/13-HODE ratio to less than 1. A higher 9-HODE/13-HODE ratio is indicative of auto-oxidation and unregulated lipid peroxidation, whereas a lower ratio is indicative of more balanced or controlled lipid metabolism via enzymatic pathways. While we did not obtain evidence for associations of oxylipins with WBV or LVV , baseline 9-HODE has been reported to be associated with greater white matter and thalamic neurodegeneration in RRMS [50].

Natural carotenoids have emerged as promising antioxidant agents to counteract redox and inflammatory cascades in MS [51]. A randomized, prospective study of 88 RRMS patients demonstrated that increased α-tocopherol intake reduced the odds for new and subsequent T2- and T1-lesions [52]. Higher intake of antioxidants did not reduce the risk of MS in females [53], or improve cognitive function [54]; however, the impact on oxylipins in MS has not been investigated.

We measured LC3A to assess the effect of oxylipins on autophagy and selective mitophagy and found that greater 9-HODE and 13-HODE levels were associated with lower LC3A levels. The conjugation of phosphatidylethanolamine to cytosolic LC3 or LC3-I results in the membrane-bound form, LC3-II (LC3-PE). Lipid peroxidation stimulates the preferential removal of oxidized LC3-PE by autophagy-related (ATG) protein-4, which inhibits autophagy [55]. ATG protein activity has been reported to be decreased in post-mortem human MS brain tissue [56]. Additionally, LC3A selectively binds externalized cardiolipin on the outer mitochondrial membrane of oxidatively damaged mitochondria to nucleate the formation of an autophagosome that triggers mitophagy [57]. Reduced mitophagy in the presence of oxylipins could lead to the buildup of damaged mitochondria, which might promote inflammation. To our knowledge, LC3A levels in MS patient samples have not been extensively investigated.

## 5. Conclusions

The 9-HODE/13-HODE ratio may be a useful biomarker in the assessment of MS. Targeted interventional therapies aimed at reducing auto-oxidation may improve disability progression and decrease chronic inflammation; however, further research is necessary to validate their effects, if any, on MS outcomes.

## Figures and Tables

**Table 1:** Demographic and clinical characteristics of the study subjects.

	HC	RR-MS	P-MS	*p*-Value
Sample size	30	68	37	
Gender, Female (%)	20 (66.6%)	48 (71%)	28 (75.7%)	0.71
Age, years	46.3 (13.0)	44.7 (10.9)	56.3 (6.33)	<0.001
Body mass index, kg/m^2^	27.6 (5.38)	27.4 (5.58)	26.2 (3.95)	0.45
Race:				0.028
Caucasian	26	65	36	
African American	4	1	0	
Hispanic/Latino	0	1	0	
Asian	0	0	0	
Other	0	0	0	
Missing	0	1	1	
Disease duration, years	-	13.6 (9.01)	22.2 (10.6)	<0.001
EDSS	-	2.0 (1.5–3.0)	5.0 (3.5–6.5)	<0.001
Disease-modifying treatments:	-			0.045
No treatment				
Interferon		14	1	
Glatiramer acetate		38	22	
Other		14	13	
		2	1	
Oxylipins, ng/mL:				
9-HODE	126	178	235	0.15
13-HODE	205	266	283	0.45
9-HODE/13-HODE	0.463	0.669	0.871	<0.001
9-HpODE	7010	7260	8930	0.68
13-HpODE	2900	2830	3400	0.65
13-HOTE	258	190	230	0.6
12-HEPE	180	282	337	0.14
5-HETE	5.04	8.79	9.30	0.14
12-HETE	502	539	563	0.66
12-HpETE	37.2	47.9	52.2	0.52

**Table 2:** Associations of oxylipins with MS disease status (HC, RR, or PMS), EDSS, and sNfL. The regression slope (β) in the RR and PMS groups (β_RR_ and β_PMS_), generalized eta-squared effect size (*η*^2^), and *p*-value are shown.

	Dependent Variables		
	MS Disease Course (β_RR_, β_PMS_) *η*^2^ (*p*-Value)	EDSS β *η*^2^ (*p*-Value)	sNFL β *η*^2^ (*p*-Value)
9-HODE	(0.111, 0.496) 0.040 (0.072)	0.378 0.038 (0.074)	0 <0.001 (0.94)
13-HODE	(0.093, −0.017) 0.004 (0.77)	0.117 0.002 (0.65)	−0.013 0.001 (0.71)
9-HODE/13-HODE	(0.128, 0.402) 0.096 (0.002)	1.19 0.094 (0.004)	00.005 (0.46)
9-HpODE	(−0.042, 0.003) 0.004 (0.78)	0.03 <0.001 (0.96)	−0.017 <0.001 (0.80)
13-HpODE	(−0.051, −0.010) 0.005 (0.74)	0.051 <0.001 (0.93)	−0.037 0.002 (0.60)
13-HOTE	(−0.135, −0.095) 0.015 (0.38)	0.023 <0.001 (0.95)	−0.013 <0.001 (0.80)
12-HEPE	(0.097, 0.127) 0.010 (0.52)	0.332 0.009 (0.40)	−0.018 0.001 (0.72)
5-HETE	(0.119, 0.157) 0.027 (0.16)	0.203 0.002 (0.69)	00.003 (0.53)
12-HETE	(0.072, 0.166) 0.017 (0.35)	−0.412 0.010 (0.36)	−0.029 0.003 (0.58)
12-HpETE	(0.023, 0.039) <0.001 (0.94)	0.542 0.022 (0.17)	−0.007 <0.001 (0.89)

**Table 3:** Associations of oxidized lipid products with antioxidant micronutrients. The regression slope (β), generalized eta-squared effect size (*η*^2^), and *p*-value are shown.

	α-Carotene	β-Carotene	α-Tocopherol	δ-Tocopherol	γ-Tocopherol	β-Cryptoxanthin	Lutein/Zeaxanthin	Lycopene
9-HODE	−0.018 0.003 (0.59)	−0.022 0.004 (0.49)	0.013 0.007 (0.36)	0.008 0.003 (0.74)	0.069 0.056 (0.009)	−0.048 0.028 (0.068)	−0.016 0.006 (0.41)	−0.009 0.002 (0.60)
13-HODE	−0.004 <0.001 (0.92)	−0.007 <0.001 (0.85)	0.031 0.026 (0.079)	0.015 0.004 (0.70)	0.096 0.068 (0.004)	−0.030 0.007 (0.37)	0.010 0.001 (0.69)	0.005 <0.001 (0.81)
9-HODE/13-HODE	−0.061 0.007 (0.36)	−0.067 0.010 (0.28)	−0.027 0.008 (0.34)	0.008 <0.001 (0.87)	0.033 0.003 (0.53)	−0.114 0.040 (0.028)	−0.088 0.043 (0.023)	−0.050 0.017 (0.16)
9-HpODE	0.036 0.002 (0.67)	0.066 0.006 (0.42)	0.070 0.031 (0.053)	0.068 0.033 (0.23)	0.140 0.035 (0.039)	−0.056 0.006 (0.41)	0.011 <0.001 (0.82)	0.001 <0.001 (0.98)
13-HpODE	0.032 0.001 (0.71)	0.058 0.004 (0.48)	0.077 0.037 (0.035)	0.079 0.043 (0.17)	0.127 0.028 (0.066)	−0.068 0.008 (0.32)	0.009 <0.001 (0.86)	0.005 <0.001 (0.91)
13-HOTE	0.150 0.047 (0.018)	0.132 0.039 (0.030)	0.066 0.047 (0.017)	0.067 0.043 (0.17)	−0.034 0.004 (0.52)	0.094 0.028 (0.067)	0.085 0.041 (0.026)	0.067 0.030 (0.057)
12-HEPE	−0.044 0.004 (0.47)	<0.001 <0.001 (0.99)	0.031 0.011 (0.25)	−0.008 <0.001 (0.86)	0.157 0.083 (0.001)	−0.081 0.023 (0.100)	−0.030 0.005 (0.42)	−0.001 <0.001 (0.97)
5-HETE	−0.090 0.010 (0.28)	−0.112 0.017 (0.16)	0.012 0.001 (0.74)	−0.031 0.008 (0.55)	0.260 0.123 (<0.001)	−0.150 0.042 (0.024)	−0.065 0.014 (0.19)	−0.044 0.008 (0.34)
12-HETE	−0.008 <0.001 (0.90)	−0.032 0.002 (0.60)	0.010 0.005 (0.64)	−0.100 0.054 (0.13)	0.037 0.004 (0.48)	−0.014 <0.001 (0.79)	0.024 0.003 (0.54)	−0.007 <0.001 (0.85)
12-HpETE	−0.087 0.016 (0.17)	−0.060 0.008 (0.33)	0.021 0.005 (0.46)	−0.009 <0.001 (0.84)	**0.218** **0.148 (<0.001)**	−0.096 0.029 (0.060)	−0.037 0.008 (0.34)	0.007 <0.001 (0.84)

**Table 4:** Associations of oxidized lipid products with triglyceride-rich lipoprotein (TRL) particle subsets. The regression slope (β), generalized eta-squared effect size (*η*^2^), and *p*-value are shown.

	TRLP Total	VL-TRLP	L-TRLP	M-TRLP	S-TRLP	VS-TRLP (IDL)	ApoC-II
9-HODE	0.091 0.177 (<0.001)	0.055 0.011 (0.42)	0.189 0.049 (0.110)	0.250 0.138 (0.004)	0.019 0.003 (0.69)	0.187 0.178 (<0.001)	0.044 0.055 (0.030)
13-HODE	0.073 0.089 (0.022)	0.050 0.007 (0.53)	0.201 0.041 (0.14)	0.342 0.197 (<0.001)	0.025 0.004 (0.65)	0.131 0.066 (0.049)	0.058 0.065 (0.019)
9-HODE/13-HODE	0.119 0.089 (0.022)	0.060 0.004 (0.64)	0.137 0.007 (0.540)	−0.037 <0.001 (0.82)	<0.001 <0.001 (1.00)	0.300 0.133 (0.005)	0.018 0.002 (0.65)
9-HpODE	0.098 0.027 (0.21)	−0.025 <0.001 (0.90)	0.285 0.015 (0.380)	0.466 0.063 (0.055)	0.249 0.065 (0.051)	−0.007 <0.001 (0.97)	0.099 0.046 (0.050)
13-HpODE	0.107 0.031 (0.180)	−0.030 <0.001 (0.88)	0.411 0.031 (0.210)	0.488 0.067 (0.048)	0.260 0.069 (0.045)	0.027 <0.001 (0.87)	0.097 0.041 (0.062)
13-HOTE	−0.039 0.011 (0.42)	−0.082 0.009 (0.48)	0.110 0.006 (0.580)	0.162 0.020 (0.28)	0.148 0.060 (0.061)	−0.152 0.041 (0.12)	−0.014 0.002 (0.70)
12-HEPE	0.147 0.126 (0.006)	0.043 0.002 (0.75)	0.023 <0.001 (0.920)	0.250 0.038 (0.140)	0.045 0.004 (0.62)	0.221 0.068 (0.046)	0.069 0.039 (0.069)
5-HETE	0.211 0.116 (0.008)	−0.028 <0.001 (0.89)	0.178 0.006 (0.600)	0.507 0.069 (0.044)	0.062 0.004 (0.65)	0.363 0.081 (0.029)	0.134 0.089 (0.005)
12-HETE	−0.050 0.015 (0.35)	0.067 0.005 (0.61)	−0.256 0.027 (0.240)	−0.116 0.008 (0.49)	−0.011 <0.001 (0.900)	−0.070 0.007 (0.53)	0.021 0.003 (0.63)
12-HpETE	0.163 0.165 (0.001)	−0.003 <0.001 (0.98)	0.216 0.019 (0.32)	0.341 0.074 (0.037)	0.080 0.015 (0.36)	0.197 0.057 (0.069)	0.106 0.092 (0.005)

**Table 5:** Associations of oxidized lipid products with biomarkers of chronic inflammation, mitophagy, and autoxidation. The regression slope (β), generalized eta-squared effect size (*η*^2^), and *p*-value are shown.

	CRP	GlycA	LC3A	7-KC
9-HODE	0.168 0.063 (0.012)	0.019 0.055 (0.036)	−0.028 0.035 (0.041)	0.076 0.051 (0.025)
13-HODE	0.195 0.053 (0.021)	0.028 0.086 (0.008)	−0.049 0.070 (0.004)	0.097 0.053 (0.022)
9-HODE/13-HODE	0.166 0.017 (0.20)	−0.002 <0.001 (0.89)	0.014 0.002 (0.61)	0.050 0.006 (0.45)
9-HpODE	0.182 0.011 (0.31)	0.044 0.044 (0.062)	−0.122 0.108 (<0.001)	0.191 0.048 (0.031)
13-HpODE	0.173 0.009 (0.34)	0.041 0.037 (0.087)	−0.122 0.103 (<0.001)	0.189 0.046 (0.034)

## Data Availability

The data that support the findings of this study are available upon reasonable request from the principal investigator of the clinical study (Dr. Robert Zivadinov). The data are not publicly available due to containing information that could compromise the privacy of research participants.

## Supplementary Materials

The following supporting information can be downloaded at https://www.mdpi.com/article/10.3390/antiox15010102/s1. Table S1. Associations of oxidized lipid products with low-density lipoprotein (LDL) particle subsets. Table S2. Associations of oxidized lipid products with high-density lipoprotein (HDL) particle distributions. Figure S1. Figures (A,B) show the associations of the cholesterol autoxidation product 7-ketocholesterol for the lowest, middle, and highest tertiles of 9-HODE and 13-HODE, respectively, in HC (salmon bars), RRMS (green bars), and PMS (blue bars).
