# Supplementary material for: Oxylipin Biomarkers of Auto-Oxidation Are Associated with Antioxidant Micronutrients and Multiple Sclerosis Disability
Source: Antioxidants (Basel). 2026 Jan 13;15(1):102. doi: 10.3390/antiox15010102 (PMC12838321; doi:10.3390/antiox15010102)
Supplement: Supplementary file 1 [file antioxidants-15-00102-s001.zip › antioxidants-4055269-supplementary.pdf]

SUPPLEMENTARY FILE

Oxylipin Biomarkers of Auto-Oxidation Are Associated with Antioxidant Micronutrients and Multiple Sclerosis Disability

Taylor R. Wicks [1], Anna Wolska [2], Diala Ghazal [3], Irina Shalaurova [4], Bianca Weinstock-Guttman [5], Richard W. Browne [3], Alan T. Remaley [2], Robert Zivadinov [6,7] and Murali Ramanathan [1,4,*]

[1] Department of Pharmaceutical Sciences, University at Buffalo, The State University of New York, Buffalo, NY 14203, USA
[2] Lipoprotein Metabolism Laboratory, National Heart, Lung, and Blood Institute, National Institutes of Health, Bethesda, MD 20892, USA
[3] Biotechnical and Clinical Laboratory Sciences, University at Buffalo, The State University of New York, Buffalo, NY 14203, USA
[4] LabCorp Diagnostics, Morrisville, NC 27560, USA
[5] Jacobs Multiple Sclerosis Center, Department of Neurology, Jacobs School of Medicine and Biomedical Sciences, University at Buffalo, The State University of New York, Buffalo, NY 14203, USA
[6] Buffalo Neuroimaging Analysis Center, Department of Neurology, Jacobs School of Medicine and Biomedical Sciences, University at Buffalo, The State University of New York, Buffalo, NY 14203, USA
[7] Center for Biomedical Imaging, University at Buffalo, The State University of New York, Buffalo, NY 14203, USA
* Correspondence: murali@buffalo.edu; Tel.: +1-(716)-645-4846

Table S1. Associations of oxidized lipid products with low-density lipoprotein (LDL) particle subsets. The regression slope (β), generalized eta-squared effect size (η^2), and *p*-value are shown.

	LDLP Total	L-LDLP	M-LDLP	S-LDLP	ApoB
**9-HODE**	**0.038**<br>**0.084 (0.025)**	-0.014<br>0.002 (0.74)	-0.022<br>0.002 (0.77)	0.073<br>0.119 (0.007)	0.023<br>0.032 (0.12)
**13-HODE**	**0.042**<br>**0.072 (0.039)**	-0.014<br>0.001 (0.78)	-0.006<br><0.001 (0.94)	0.061<br>0.064 (0.052)	**0.036**<br>**0.054 (0.047)**
**9-HODE/13-HODE**	0.025<br>0.010 (0.45)	-0.012<br><0.001 (0.88)	-0.057<br>0.004 (0.67)	0.086<br>0.05 (0.085)	-0.005<br><0.001 (0.86)
**9-HpODE**	**0.101**<br>**0.072 (0.038)**	0.123<br>0.019 (0.29)	-0.070<br>0.003 (0.72)	0.079<br>0.018 (0.30)	**0.080**<br>**0.056 (0.042)**
**13-HpODE**	**0.099**<br>**0.067 (0.046)**	0.116<br>0.017 (0.33)	-0.083<br>0.004 (0.67)	0.086<br>0.021 (0.27)	**0.079**<br>**0.053 (0.049)**
**13-HOTE**	0.014<br>0.004 (0.65)	0.009<br><0.001 (0.90)	-0.023<br><0.001 (0.85)	0.010<br><0.001 (0.84)	-0.007<br><0.001 (0.80)
**12-HEPE**	**0.118**<br>**0.204 (<0.001)**	0.118<br>0.037 (0.14)	-0.057<br>0.004 (0.67)	0.140<br>0.12 (0.007)	**0.069**<br>**0.08 (0.014)**
**5-HETE**	**0.139**<br>**0.124 (0.006)**	0.077<br>0.007 (0.53)	**-0.434**<br>**0.088 (0.034)**	0.258<br>0.179 (<0.001)	**0.103**<br>**0.099 (0.006)**
**12-HETE**	-0.024<br>0.008 (0.49)	-0.098<br>0.026 (0.22)	0.029<br><0.001 (0.83)	-<0.001<br><0.001 (0.99)	-0.045<br>0.028 (0.15)
**12-HpETE**	**0.116**<br>**0.209 (<0.001)**	0.125<br>0.044 (0.11)	-0.105<br>0.012 (0.44)	0.141<br>0.128 (0.005)	**0.095**<br>**0.152 (<0.001)**

Table S2. Associations of oxidized lipid products with high-density lipoprotein (HDL) particle distributions. The regression slope (β), generalized eta-squared effect size (η^2), and *p*-value are shown.

	HDLP Total	L-HDLP	M-HDLP	S-HDLP	Apo-AI	Apo-AII
**9-HODE**	-0.014<br>0.037 (0.086)	**-0.089**<br>**0.065 (0.023)**	-0.018<br>0.011 (0.35)	0.001<br><0.001 (0.91)	-0.013<br>0.035 (0.061)	-0.007<br>0.007 (0.42)
**13-HODE**	-0.016<br>0.034 (0.10)	**-0.094**<br>**0.049 (0.049)**	-0.008<br>0.001 (0.75)	-0.005<br>0.002 (0.72)	-0.011<br>0.018 (0.18)	-0.004<br>0.001 (0.74)
**9-HODE/13-HODE**	-0.011<br>0.006 (0.49)	-0.090<br>0.018 (0.23)	-0.047<br>0.02 (0.20)	0.016<br>0.008 (0.44)	-0.020<br>0.024 (0.12)	-0.017<br>0.011 (0.29)
**9-HpODE**	-0.022<br>0.014 (0.30)	-0.046<br>0.002 (0.66)	-0.041<br>0.008 (0.43)	-0.011<br>0.002 (0.70)	-0.011<br>0.004 (0.54)	-0.018<br>0.006 (0.43)
**13-HpODE**	-0.021<br>0.012 (0.33)	-0.086<br>0.008 (0.42)	-0.027<br>0.003 (0.60)	-0.006<br><0.001 (0.83)	-0.012<br>0.005 (0.50)	-0.015<br>0.004 (0.51)
**13-HOTE**	-0.003<br><0.001 (0.82)	-0.019<br><0.001 (0.79)	-0.031<br>0.01 (0.37)	0.012<br>0.005 (0.53)	-0.003<br><0.001 (0.81)	-0.004<br><0.010 (0.80)
**12-HEPE**	0.007<br>0.003 (0.64)	-0.018<br><0.001 (0.81)	-0.164<br>0.002 (0.66)	0.027<br>0.022 (0.19)	0.004<br><0.001 (0.76)	-0.010<br>0.003 (0.56)
**5-HETE**	-0.018<br>0.008 (0.44)	-0.131<br>0.016 (0.26)	-0.183<br>0.001 (0.75)	0.003<br><0.001 (0.92)	-0.008<br>0.002 (0.64)	-0.005<br><0.001 (0.84)
**12-HETE**	0.017<br>0.018 (0.24)	0.103<br>0.028 (0.14)	0.062<br>0.043 (0.065)	0.013<br>0.006 (0.49)	0.005<br>0.002 (0.68)	0.011<br>0.004 (0.52)
**12-HpETE**	-0.003<br>0.001 (0.84)	-0.067<br>0.01 (0.38)	-0.003<br><0.001 (0.94)	0.014<br>0.006 (0.51)	-0.004<br>0.001 (0.74)	0.004<br><0.001 (0.98)

FIGURE LEGENDS

Figure S1. Figures S1A and S1B show the associations of the cholesterol autoxidation product 7-ketocholesterol for the lowest, middle, and highest tertiles of 9-HODE and 13-HODE, respectively, in HC (salmon bars), RRMS (green bars), and PMS (blue bars). Figures S1C and S1D show the associations of the mitophagy biomarker LC3A (in ng/ml) for the lowest, middle, and highest tertiles of 9-HODE and 13-HODE, respectively, in HC (salmon bars), RRMS (green bars), and PMS (blue bars). The bars are mean values, and the error bars are standard errors.

FIGURE S1

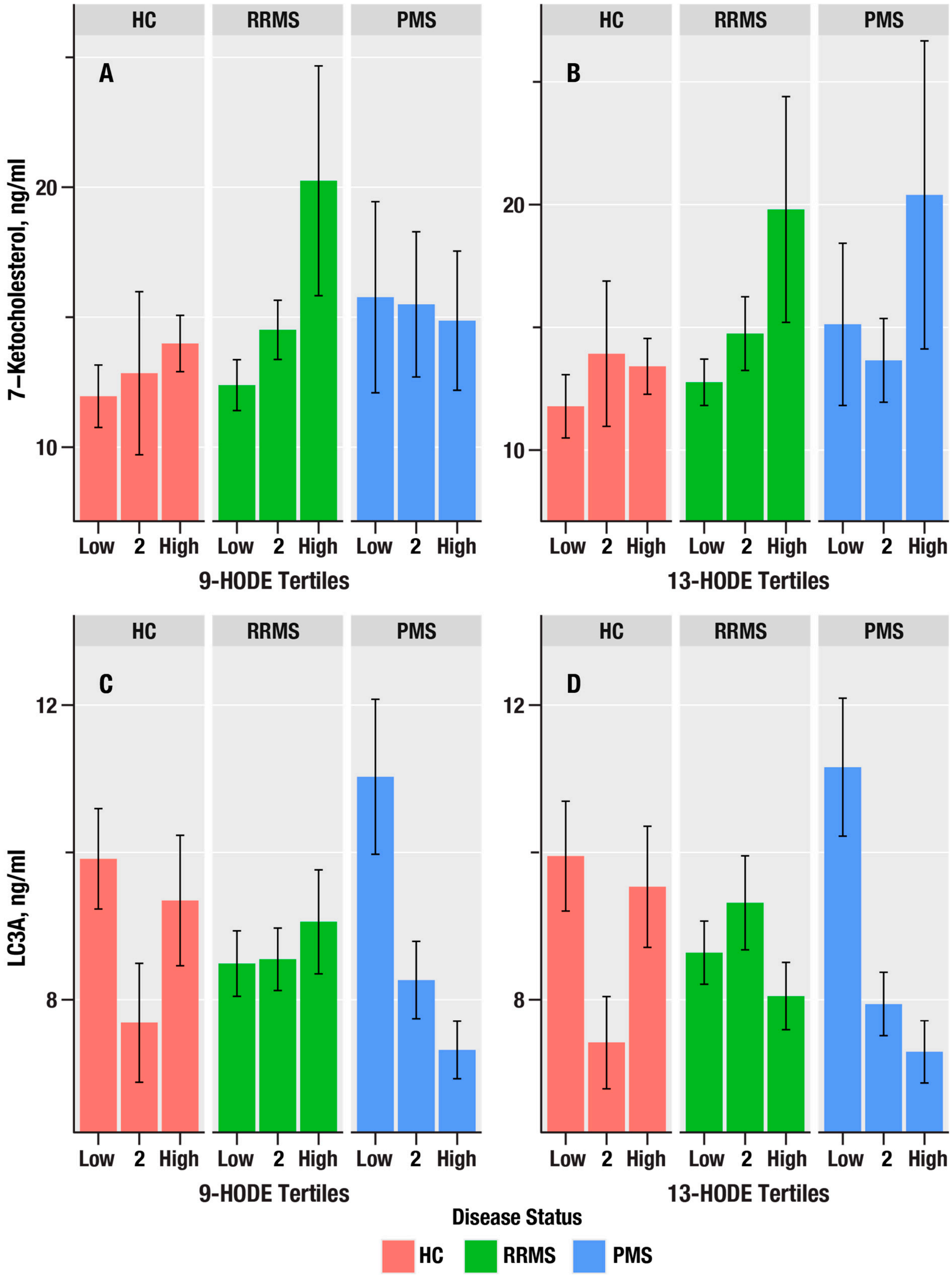

A
B
C
D
HC
RRMS
PMS
7−Ketocholesterol, ng/ml
LC3A, ng/ml
10
20
8
12
Low
2
High
9-HODE Tertiles
13-HODE Tertiles
Disease Status
HC
RRMS
PMS
